# Who’s afraid of genetic tests?: An assessment of Singapore’s public attitudes and changes in attitudes after taking a genetic test

**DOI:** 10.1186/s12910-022-00744-5

**Published:** 2022-01-26

**Authors:** Ross Cheung, Shreshtha Jolly, Manoj Vimal, Hie Lim Kim, Ian McGonigle

**Affiliations:** 1 School of Social Sciences, Nanyang Technological University, 48 Nanyang Avenue, Singapore, Singapore 639818; 2 School of Biological Sciences, Nanyang Technological University, 60 Nanyang Drive, Singapore, Singapore 637551; 3 Asian School of the Environment, Singapore Center for Environmental Life Sciences Engineering, Nanyang Technological University, 50 Nanyang Ave, Singapore, Singapore 637459

**Keywords:** Bioethics, Genetic testing, Precision medicine, Public attitudes, Singapore

## Abstract

**Background:**

As a consequence of precision medicine initiatives, genomic technologies have rapidly spread around the world, raising questions about genetic privacy and the ethics of data sharing. Previous scholarship in bioethics and science and technology studies has made clear that different nations have varying expectations about trust, transparency, and public reason in relation to emerging technologies and their governance. The key aims of this article are to assess genetic literacy, perceptions of genetic testing, privacy concerns, and governing norms amongst the Singapore population by collecting surveys.

**Methods:**

This study investigated genetic literacy and broad public attitudes toward genetic tests in Singapore with an online public survey (n = 560). To assess potential changes in attitudes following receipt of results from a genetic test, we also surveyed undergraduate students who underwent a genetic screen as part of a university class before and after they received their test results (n = 25).

**Results:**

Public participants showed broad support for the use of genetic tests; scored an average of 48.9% in genetic literacy; and expressed privacy concerns over data sharing and a desire for control over their genetic data. After taking a genetic test and receiving genetic test results, students reported less fear of genetic tests while other attitudes did not change significantly.

**Conclusion:**

These findings highlight the potential of genetic education and active engagement with genetic testing to increase support and participation in genomic projects, PM, and biobanking initiatives; and they suggest that data privacy protections could potentially reduce discrimination by giving participants control over who can access their data. More specifically, these findings and the dataset we provide may be helpful in formulating culturally sensitive education programs and regulations concerning genomic technologies and data privacy.

**Supplementary Information:**

The online version contains supplementary material available at 10.1186/s12910-022-00744-5.

## Background

In recent years, genome technologies have rapidly fallen in cost and massively increased in speed, leading to their global expansion and yielding unprecedented amounts of sensitive genomic information [1–3]. The emerging model of precision medicine (PM) necessitates large-scale population-based genome sequence data [4–7] and states and private companies have launched genome programs, and are generating massive amounts of sensitive data regarding disease susceptibility [8]. Although genome projects promise benefits in the form of future therapies, they also raise several concerns. Beyond the technical challenges of sequencing and data storage, there is also a range of social and cultural concerns [9–12] such as citizens’ rights, risk status, informed consent, data privacy, social stigma, the commercial exploitation of genomic data, and benefit-sharing [13–18].

Several studies have highlighted the importance of genetic literacy with regard to public attitudes toward participation in genomics research. A recent study of Qatari citizens [19], for example, found that willingness to participate in genome projects is linked with basic literacy in genetics, prior experience with genetic testing, and a family history of genetic diseases. A UK-based study [20] found that genetics education, familiarity with science, technology, engineering, math or medicine (STEMM), and gender were all significant variables influencing data sharing preferences. Other studies have identified ethnicity, culture and religion as pertinent factors affecting attitudes and participation in genomics research.

So far, however, there are few studies of Southeast Asians’ views regarding genetic testing. Two recent focus group discussion (FGD)-based studies [21, 22] of Singapore citizens’ and Permanent Residents’ (PRs) concerns about genomic and medical data sharing found support for data sharing with data security and de-identification measures; and the study reported participants’ broad recognition of the societal value of genetics research for PM [21]. Participants also favoured a data-sharing oversight body that could strengthen the public trust [22]. Another Singapore-based study that explored the attitudes and preferences of cancer patients and clinicians towards data usage and data-sharing for research [23] found cancer patients had limited knowledge about cancer and genetics as well as the procedures and safeguards of informed consent. Both patients and clinicians felt personal identifiers should be de-linked from stored data before sharing with third parties. According to some clinicians’ views, patients would likely give consent if they are assured that their data will be kept confidentially and in keeping with the existing local laws and guidelines. Other Singapore-based clinical studies emphasized the influence of counselling and family support in affecting patients’ decision to undergo genetic testing [24] or share results with family [25].

Thus far, however, Singapore’s lay public attitudes and knowledge of genetics and genetic privacy have not been assessed. The key aims of this article are to assess genetic literacy, perceptions of genetic testing, privacy concerns, and governing norms amongst the Singapore population by collecting surveys. The secondary aims are to identify how these categories vary across demographic lines and to determine possible changes in attitude after receiving results of a genetic test. In doing so, we aim to yield a data set that could facilitate the formation of tailored bioethical guidelines for genetic testing and personal data protection.

## Methods

This study consists of two surveys. The first survey addressed attitudes and knowledge amongst the general public of Singapore aged 21 years or older. Participants were required to be Singapore citizens, PRs, or long-term residents. The second survey addressed potential changes in attitude following experience with genetic testing and participants were university students enrolled in a biology module on genetics and evolution at Nanyang Technological University (NTU). As part of the educational component of the module, students were offered a personal genetic test kit and participation was voluntary. Students who participated in the genetic test were invited to partake in a survey on attitudes and beliefs before and after they received the results of the genetic test. Prior informed consent for participation in both surveys was obtained from the respondents and both surveys were conducted online in Singapore in line with the project’s approval by NTU-Institutional Review Board (NTU- IRB-2020-07-056).

### Public survey of knowledge and attitudes regarding genetic testing

A literature review was performed for research papers that assessed attitudes and perceptions of the general public toward genetic testing and genome projects, targeting standard databases using keywords: “attitudes”, “perception”, “concerns”, “public”, “society”, “genetic testing”, “biodata sharing”, “genomics” and “genomic data.” Thirteen articles published from 2009 to 2019 were selected [13–20, 26–30] from which survey questions were assessed and compiled into a question bank and categorized according to themes of “sociological factors”, “literacy”, “perceptions”, “privacy concern” and “governance”, and paraphrased appropriately to suit the Singaporean context. Additional questions specific to COVID-19 and racial discrimination were also synthesized to explore beliefs about the relationships between COVID-19 and ethnic diversity. To measure attitudes, most survey questions involved a 5-point Likert type scale with “−2” corresponding to strongly disagree, “−1” to somewhat disagree, “0” to neutral, “+1” to somewhat agree, “+2” to strongly agree. Other questions involved “yes/no” or “true/false/don’t know” responses (See Additional file 1). To avoid leading participants’ responses toward agreement, some questions were framed negatively and scores were negated for such questions. Qualtrics XM was contracted to recruit a sample representative of Singapore society, following ethical safeguards, including informed consent, anonymity and confidentiality. Based on the answers to the survey questions of demographic profile, we categorized participants into varying groups such as gender, age, and ethnicity. For each group of quantifiable questions, the mean score for each demographic group was calculated. Mean scores were tested for differences across the groups within each demographic category using two-tailed t-tests for two groups and one-way ANOVA for more than two groups.

### Student survey of changes in attitudes following a genetic test

Pre-test and post-test surveys were created using Microsoft Forms with 48 and 45 questions respectively, using the same scoring scale as the public survey described above (see Additional file 1). Students were offered a personal genetic test kit (Genoplan, Singapore) which informs on the risk of various health conditions, including cancer, metabolic diseases, brain health, and drug responses. Participants were given a unique identifier to access the survey. After undertaking the pre-test survey, six weeks later participants received their genetic test results and were immediately provided access to the survey link for the post-test survey accessible with their serial key. Survey data were analysed as above.

## Results

### Public survey of knowledge and attitudes regarding genetic testing

#### Participant demographics

We received responses from 560 participants, both male (57.9%) and female (42.1%), in the range of 21–39 (49.2%), 40–59 (42.1%) and above 60 (8.5%) years (See Table 1). The group included participants who self-identified as Chinese (81.8%), Malay (7.9%) and Indian (5.7%) as well as “Others” (4.6%), such as Eurasian, European or mixed-race. This sample fairly represents Singapore society’s ethnic, racial, and religious demographics.

**Table 1:** Singapore public survey data

	%	N	Literacy			Perception of genetic testing			Data privacy concern		
			Mean	SD	*p*	Mean	SD	*p*	Mean	SD	*p*
*Gender*					(t-test) 0.977			(t-test) 0.397			(t-test) 0.147
Male	57.9%	324	48.8%	0.50		0.77	0.95		0.61	1.20	
Female	42.1%	236	48.9%	0.50		0.73	0.95		0.66	1.20	
*Age*					(ANOVA) *			(ANOVA) 0.641			(ANOVA) 0.918
21–39	49.3%	276	51.2%	0.50		0.77	0.95		0.64	1.19	
40–59	42.1%	236	47.0%	0.50		0.73	0.97		0.64	1.21	
60 and higher	8.6%	48	44.7%	0.50		0.79	0.86		0.61	1.20	
*Ethnicity*					(ANOVA) 0.366			(ANOVA) 0.400			(ANOVA) 0.387
Chinese	81.8%	458	48.5%	0.50		0.76	0.93		0.64	1.19	
Malay	7.9%	44	49.1%	0.50		0.65	1.06		0.56	1.23	
Indian	5.7%	32	48.5%	0.50		0.85	1.04		0.60	1.28	
Others	4.6%	26	55.0%	0.50		0.71	1.01		0.75	1.24	
*Annual household income*					(ANOVA) **			(ANOVA) *			(ANOVA) 0.341
Up to SGD 30,000	14.1%	79	43.7%	0.50		0.59	0.94		0.68	1.14	
SGD 30,000–59,999	18.6%	104	48.7%	0.50		0.80	0.95		0.62	1.21	
SGD 60,000–89,999	23.4%	131	48.3%	0.50		0.75	0.92		0.59	1.18	
SGD 90,000–149,999	31.4%	176	49.0%	0.50		0.74	0.98		0.67	1.19	
SGD 150,000 and more	12.5%	70	55.5%	0.50		0.92	0.91		0.60	1.29	
*Genetics education*					(t-test) **			(t-test) *			(t-test) 0.490
No Genetics education at all	67.3%	377	45.2%	0.50		0.71	0.95		0.63	1.20	
With Genetics education	32.7%	183	56.3%	0.50		0.84	0.93		0.65	1.20	
*Education attainment*					(ANOVA) **			(ANOVA) 0.376			(ANOVA) 0.936
Less than Bachelor's Degree	34.8%	195	44.7%	0.50		0.71	0.93		0.62	1.19	
Bachelor's Degree	53.0%	297	50.1%	0.50		0.78	0.95		0.64	1.20	
Master's Degree	11.4%	64	55.3%	0.50		0.78	1.00		0.62	1.24	
PhD, MD, JD or equivalent	0.7%	4	52.9%	0.50		0.56	0.92		0.68	1.10	
*Patients*											
Genetic diseases patients	8.0%	45	50.7%	0.50	(t-test) 0.418	0.61	1.02	(t-test) *	0.44	1.16	(t-test) **
Non-genetic diseases participants	92.0%	515	48.7%	0.50		0.77	0.94		0.65	1.20	
COVID patients	1.4%	8	62.5%	0.49	(t-test) *	0.76	0.93	(t-test) 0.875	0.45	1.09	(t-test) 0.197
Non-COVID participants	98.6%	552	48.7%	0.50		0.75	0.95		0.64	1.20	
Total sample	100%	560	48.9%	0.50		0.75	0.95		0.63	1.20	

^*^Indicates a *p* value from a t-test or ANOVA < 0.05;

^**^Indicates a *p* value of a t-test or ANOVA < 0.001

#### Literacy

The mean participant score was 48.9% across the set of 17 questions (See Tables 1, 2). For basic literacy questions, most answered correctly: for example, 80.5% of participants were aware that we have more genes in common with our siblings than our cousins. Similarly, 78% of respondents were aware that some genetic diseases appear later in adult life rather than only appearing earlier in childhood. However, a large proportion of the participants gave the wrong answers for some questions, for example, “The sex of the baby is determined by the father” (38.6% incorrect; See Table 2).

**Table 2:** Genetic Literacy Questions for the Singapore Public Survey

		True	False	Don’t know
1	If two people are from the same ethnicity, they will be more genetically similar to each other than two people from different ethnicities	**65.5%**	17.5%	17.0%
2	You have more genes in common with your brother or sister than with your cousins	**80.5%**	9.1%	10.4%
3	People share more genes with their paternal cousins than their maternal cousins	33.9%	32.3%	**33.8%**
4	The sex of the baby is determined by the father	47.9%	**38.6%**	13.6%
5	Every trait is controlled by a specific single gene. (For example: Height is controlled by a height gene; Eye colour is controlled an eye gene)	53.8%	**20.7%**	25.5%
6	We can only say that a disease is genetic if it has affected more than one family member	**55.5%**	27.1%	17.3%
7	Some genetic diseases appear later in adult life rather than appearing earlier in childhood	**78.0%**	10.2%	11.8%
8	Health habits affect the severity of some genetic diseases	**75.2%**	10.5%	14.3%
9	Genes come in pairs, with one copy inherited from each parent	**53.0%**	16.1%	30.9%
10	The chromosomes of men and women are similar except for one pair	**47.1%**	14.5%	38.4%
11	For some disorders to be inherited, a mutation must come from both parents	**34.8%**	33.2%	32.0%
12	Males and females have the same number of chromosomes	**38.4%**	32.0%	29.6%
13	A gene is a disease	9.6%	**75.4%**	15.0%
14	The carrier of a disease gene may be completely healthy	**62.3%**	12.1%	25.5%

		Correct answer	Wrong answers	Don't know
15	How many pairs of chromosomes do humans have?	**41.8%**	19.8%	38.4%
16	In DNA, the Adenine nucleotide bonds with which nucleotide to form a base pair?	**18.4%**	16.6%	65.0%
17	Approximately how many protein-encoding genes do humans have?	**11.4%**	25.4%	63.2%

Correct answers are bolded

Literacy questions show significant differences in mean score across various categories of demographic groups, such as age and income. Participants younger than 40 years of age scored higher in genetic literacy (51.2%) when compared with those aged 60 and older (44.7% correct, *p* < 0.01 ANOVA). Participants with annual household income greater than SGD 150,000 scored more questions correctly (55.5% correct) than the lower-income (< 30,000 SGD) groups (43.7% correct, *p* < 0.001 ANOVA). Participants who studied genetics as part of biology education scored higher in genetic literacy (56.3%) than the participants who did not study genetics (45.2%, *p* < 0.001 t-test). Participants with lower educational attainment (less than Bachelor’s degree) scored lower in literacy (44.7%) than more highly educated groups (> 50.1%, *p* < 0.001 ANOVA). Within our sample (n = 560), 45 participants had previously been diagnosed with a genetic disease. This group had higher literacy (50.7%) than literacy in non-diagnosed participants (48.7%, *p* < 0.05 t-test). The eight participants that reported being previously diagnosed with COVID-19 also scored higher (62.5%) for genetic literacy than non-COVID participants (48.7%, *p* < 0.05 t-test).

#### Perception of genetic testing

Most participants agreed (62.6%) that genetic testing can improve the quality of life and health. While 34.3% of the participants claimed confidence in understanding the relevant information about results from a genetic test, 65.7% were neutral. 45.9% of participants agreed that genetic testing does more good than harm while 13% disagreed. 47.1% disagreed that genetic testing is tampering with the will of God although 38.7% agreed (of whom, 39.2% were Muslims, 21.4% Christians, 18.5% Taoist, 17.2% Buddhist, and 9.5% Hindus).

The mean score for perceptions towards genetic testing across all questions was derived to be 0.75 (SD 0.95) in a range of **− **2 to + 2 indicating a mean positive perception. Participants who had earlier been diagnosed with a genetic disease held less positive perceptions of genetic testing, with a score of 0.61 compared to the non-diagnosed participants (0.77, *p* < 0.05 t-test) (See Table 1). Participants who studied genetics as part of biology education at university, however, had more positive perceptions (0.84) than the participants who never studied genetics (0.71, *p* < 0.01 t-test). The participants in the highest income group (> SGD 150,000) scored higher for positive perceptions (0.92, *p* < 0.01 ANOVA).

51.4% of participants felt that by donating their samples to a genetic database they would be helping future generations and 54.1% felt that taking part in genetic databases could lead to better medical treatments. Participants broadly approved the use of genetic testing to know their genetic risk for a wide range of medical conditions: 79.6% approved of the use of genetic testing for optimizing medical treatment and a majority approved of the use of genetic testing (76.6% agreed) to determine the likelihood of future diseases, with the greatest interest in kidney diseases, diabetes, heart disease, Alzheimer’s and rare diseases.

#### Data privacy concern

Overall, participants expressed concerns over privacy issues with 77.5% agreeing they would worry if their genetic information were shared, with 6.8% disagreeing and 15.7% neutral. Most (81.6%) wanted to know if their health information would subsequently be shared with insurance companies if they opt for genetic testing, and 64.4% of participants agreed that they would worry that research they did not consent to would be done. In terms of legal protections against racial discrimination in the context of genomics, a majority disagreed (63.9%) that if new scientific findings show ethnic differences in COVID-19 susceptibility this information should be kept secret. For genetic data privacy concerns, the mean score derived from the relevant questions was 0.63 (See Table 1), indicating that participants have broad concerns regarding the sharing of their data. The mean scores across demographic groups did not show significant difference except for one category, between the participant groups of the genetic disease diagnosed (0.44) and the undiagnosed (0.65, *p* < 0.001 t-test).

#### Governance

Regarding who should control and take ethical and legal responsibility for the samples and data in a large-scale biobank or genetic database project, 54.4% of participants agreed that people who provided their genetic samples should have control; a majority (73%) of participants agreed that researchers who conduct the biobank project should control and take ethical and legal responsibility for such projects.

Regarding benefit-sharing from large-scale genetics research, the data were unclear in delineating views. 61.7% of participants agreed that the people who provided their genetic samples should benefit from large-scale genetics research while 60% agreed that the researchers who conducted the research project or their institution also should benefit from such projects. Most participants (85.7%) expressed interest in knowing who runs the genetic database if they take a genetic test.

### Student survey of changes in attitudes following a genetic test

#### Participant demographics

25 students volunteered for the genetic testing and completed both the pre-test and post-test surveys, 84.0% of whom were ethnically Chinese and 64.0% of whom were female and all of whom were aged 18–29 years.

#### Changes in attitude after taking a genetic

Most survey items did not show a significant difference in participant responses after undertaking the genetic test. For the survey item “The idea of a genetic test frightens me,” however, a significantly higher proportion of participants disagreed after undertaking the test (*p* = 0.01 paired t-test). Also, a significantly higher proportion of participants discussed their genetic test with their families after receiving the test results (80.0% after versus 52.0% before, *p* < 0.05 McNemar test).

## Discussion

Until now, studies of public attitudes toward genetics and data sharing have focused on the issues of informed consent [28], privacy [1], discrimination [31], stigmatization [32, 33] and benefit-sharing [34] although most of these studies have been in Western contexts. This study asked similar questions of public attitudes toward large-scale genome projects but in the specific context of multi-racial Singapore. Our findings are timely given the recent proliferation of genome research in Singapore in recent years [35–37]. This novel dataset also informs on a small multi-racial state in the Asian context, albeit one that is highly developed and technologically advanced. In this regard, the findings presented here from Singapore may be comparable to other wealthy and developed Asian societies such as Japan or Korea.

In terms of genetic literacy in Singapore, we report an average genetic literacy score that may indicate a need for more public education and engagement programs on genetics and participation in PM projects. This level of literacy is similar to survey findings recently reported from Qatar [19], where 51.6% of the participants scored “high” for genetic literacy. The same study found that amongst respondents with a high level of basic literacy in genetics, 76.4% expressed willingness to participate in the Qatar Genome Programme activities, suggesting genetic literacy may be a factor affecting enrolment rates.

We followed a previous Singapore FGD-based study’s call to assess “governing norms and values” [21] and our survey represents the views of Singapore society. Our study shows that Singapore residents are concerned their genetic data could be shared with third parties, highlighting the importance of public trust and transparency in terms of data security and privacy protection. A potential policy response to alleviate such concerns might be to ensure donor control of third-party access to genetic information [38].

Another way attitudes could be shaped is through active participation in genetic testing. By demonstrating a reduction in fear of genetic tests amongst university students after they received their test results, we invite further studies to consider whether hands-on education, outreach interventions, and the consequent familiarity with genetic testing, may improve participation rates in genomic projects and PM initiatives. Indeed, our public survey findings suggest that genetics education is associated with increased support and positive perception of genomics research and genetic testing. Moreover, participants previously diagnosed with a genetic disease reported less data privacy concern, suggesting that exposure to genetic testing may reduce concerns about genetic testing and its consequences.

Indeed, some scholars suggested genetic discrimination is a possibility in Singapore in the absence of genetic non-discrimination regulations [39]. Thus far, in Singapore, the use of personal data for research is regulated under the Personal Data Protection Act and the Human Biomedical Research Act. Yet, “there are currently no laws in Singapore to protect patients against employments and insurance discrimination due to their genetic status” [23]. So far, only a few countries have passed laws to protect their citizens from discrimination arising from their genetic data. The US Health Insurance Portability and Accountability Act of 1996 [40] protects sensitive patient health information from being disclosed without patient consent; and the US Genetic Information Non-Discrimination Act of 2008 disallows the request or detrimental usage of genetic information by employers and/or insurance providers [17]. In Europe, the General Data Protection Regulation [41] addresses some of these concerns [16, 42] by giving individuals control over data through consent. Singapore could look to these contexts for guidance in drafting its genetic privacy legislation.

## Limitations and future research

As our student survey sample of 25 may not capture other significant changes in attitudes following genetic testing, we suggest further work incorporate a control survey to assess changes in attitude as a consequence of genetics education alone. Moreover, the cohort studied is one high in genetic literacy and subjects participated voluntarily and may not be representative of the wider population. Our preliminary findings also raise the question of potential unintended discriminatory effects and potential social disharmony if public education about ethnic and religious differences in relation to genomics is not addressed.

## Conclusions

In conclusion, we show broad support for the use of genetic tests in Singapore, with moderate genetic literacy in the general population, and with broad privacy concerns regarding data sharing and governance. However, after taking a genetic test and receiving genetic test results, participants reported significantly less fear of genetic tests. Together these findings highlight the potential of genetic education and active engagement with genetic testing to increase support and participation in genomic projects, PM, and biobanking initiatives; and they suggest that data privacy protections could potentially reduce discrimination by giving participants control over who can access their data. The value of this dataset lies in its cultural specificity and relevance to Singapore for the development of future PM research projects and clinical initiatives. Beyond the scholarly significance of our findings for the social study of genomic technologies in Singapore, however, our findings may also be of interest to biotech companies, clinicians, and community leaders around the world interested in developing culturally tailored bioethical protocols for genomic technologies.

## Supplementary Information

**Additional file 1**. Microsoft Excel file with raw public survey and student survey data.

## Data Availability

All data generated or analyzed during this study are included in Additional file 1. Further enquiries can be directed to the corresponding author.

## Abbreviations

FGD: Focus group discussion
NTU: Nanyang Technological University
PM: Precision medicine
PRs: Permanent residents
